# Improvement of the Survival of Human Autologous Fat Transplantation by Adipose-Derived Stem-Cells-Assisted Lipotransfer Combined with bFGF

**DOI:** 10.1155/2015/968057

**Published:** 2015-01-28

**Authors:** Aimei Jiang, Ming Li, Wenjing Duan, Yilong Dong, Yanmei Wang

**Affiliations:** ^1^The First Affiliated Hospital of Kunming Medical University, Kunming, Yunnan 650031, China; ^2^The Third Affiliated Hospital of Kunming Medical University, Kunming, Yunnan 650031, China; ^3^School of Medicine, Yunnan University, Kunming, Yunnan 650091, China

## Abstract

Adipose-derived stem cells (ASCs) transplanted along with autologous adipose tissue may improve fat graft survival; however, the efficacy of ASCs has been diluted by low vascularization. This study was designed to test the hypothesis that basic fibroblast growth factor (bFGF) may improve the effects of ASCs because it owns the property to boost angiogenesis. In the present study, human fat tissues were mixed with ASCs, ASCs plus 100 U bFGF, or medium as the control and then injected subcutaneously into immunologically compromised nude mice for 12 weeks. Our findings demonstrated that mixture with the ASCs significantly increased the weight and volume of the fat grafts compared to control grafts, and histological analysis revealed that both ASCs and ASCs plus bFGF grafts consisted predominantly of adipose tissue and had significantly less fibrosis but greater microvascular density compared with control and also grafts mixed with ASCs had a high expression of angiogenic factors. More importantly, the bFGF treated fat grafts shown elevate in survival, vascularization, and angiogenic factors expression when compared with the grafts that received ASCs alone. These results indicated that bFGF together with ASCs can enhance the efficacy of autologous fat transplantation and increase blood vessel generation involved in the benefits from bFGF.

## 1. Introduction

Autologous fat transplantation for soft tissue augmentation to reconstruct inborn or acquired tissue defects is an increasingly ideal method in the field of plastic surgery. However, the low survival rate and high reabsorption rate of the transplanted fat reduce the efficacy of this technique [1]. Thus, using better methods to improve the efficacy of fat transplantation got more attentions.

With the development in stem cell research, Matsumoto et al. described a novel technique of autologous tissue transfer [2], named cell-assisted lipotransfer (CAL). For CAL, adipose-derived stem cells (ASCs) were harvested and then transplanted along with autologous adipose tissue. ASCs can differentiate directly into adipocytes and contribute to adipose tissue regeneration, and they also secrete multiple antiapoptotic growth factors to alleviate adipocytes loss, which finally enhance the viability of fat grafts. Yoshimura et al. used CAL in patients with facial lipoatrophy and cosmetic breasts augmentation has shown an excellent result [3, 4]. However, emerging evidence found that vascularization insufficient is another obstacle for fat grafts survival including in CAL [5]. Therefore, promoting the generation of blood vessels shall be beneficial to boost the efficacy of CAL.

Basic fibroblast growth factor (bFGF, also called FGF-2) is a member of fibroblast growth factors, which may modulate stem cells self-renewal, differentiation, and survival [6]. Importantly, there were evidences to support that bFGF involved in the processes of vasculogenesis, angiogenesis, and blood vessel remodeling [7, 8] make us postulate that combined bFGF treatment with CAL may promote the efficacy of autologous fat transplantation. To investigate the hypothesis, aspirated fats treated with or without bFGF have been transplanted in immunologically compromised nude mice along with ASCs, the survival and angiogenesis have been compared in different fat grafts to evaluate the effect of bFGF, and the expression of angiogenic factor, including vascular endothelial growth factor (VEGF), platelet-derived growth factor- (PDGF-) BB, and matrix metalloproteinases 2 (MMP2), also has been evaluated to partially explain the potential mechanism involved in bFGF.

## 2. Materials and Methods

### 2.1. Materials

bFGF, type I collagenase, was purchased from Sigma-Aldrich (Saint Louis, MO, USA). Dulbecco's modified Eagle's medium (DMEM) and fetal bovine serum (FBS) were obtained from Invitrogen Corporation (Carlsbad, CA, USA). CD29, CD44, CD105, CD34, and CD45 antibody were purchased from Abcam (Abcam, Cambridge, UK). VEGF, PDGF-BB, MMP2, factor VIII, and *β*-actin antibody were purchased from Santa Cruz Biotechnology (Santa Cruz, CA, USA). The protease inhibitor mixture, BCA Protein Assay Kit, and enhanced chemiluminescence substrate kit were purchased from Pierce Biotechnology (Rockford, IL, USA). The avidin-biotin-peroxidase and DAB kits for immunohistochemical detection were obtained from Zhongshang Goldenbridge Biotechnology (Beijing, China). RIPA buffer was purchased from Beyotime Biotechnology Inc. (Shanghai, China). All other chemicals used were of the highest grade commercially available.

### 2.2. Human Tissue Sampling

We obtained liposuction aspirates from six healthy female donors (age between 35 and 42 years) undergoing liposuction of the abdomen. The participant gave her written informed consent, and the study was reviewed and approved by the institutional review board of the Kunming Medical University. From the same donor, part of the aspirated fat was processed to culture ASCs, and the remaining fat was stored at −80°C until used for transplantation [9].

### 2.3. Adipose-Derived Stem Cells Isolation and Culture

ASCs were isolated and cultured according to the method described by Hsiao et al. [10]. Briefly, adipose tissue was minced into 1 mm^3^ pieces and washed extensively with equal volumes of phosphate buffered saline (PBS) and then treated with 0.075% type I collagenase for 40 min at 37°C with gentle agitation. The collagenase was neutralized with 2 volumes of DMEM containing 10% FBS and the cell suspension was centrifuged at 1200 rpm for 10 min to remove adipocytes. The pellet was resuspended in 0.16 M NH_4_CL and incubated at room temperature for 5 min to lyse red blood cells followed by filtered through a 100 *μ*m nylon mesh to remove connective tissue debris. The cells were collected by centrifugation at 1200 rpm for 5 min and resuspended in DMEM supplemented with 10% FBS, 100 U/mL penicillin, and 100 *μ*g/mL streptomycin and cultured at 37°C in a humidified atmosphere containing 5% CO_2_. ASCs at passage 2 were used in the experiments [9]. The cells have been previously characterized as ASCs by their expression mesenchymal stem cell markers using immunohistochemistry of CD29, CD44, and CD105, but not hemopoietic lineage markers CD34 and CD45.

### 2.4. Mouse Models for Transplantation of Human Aspirated Fat

CD-1 nude mice (7-week-old) were purchased from Weitong Lihua Experimentary Animal Central (Beijing, China). Animals were housed in groups of two in a room with an artificial 12 h light/dark schedule and had free access to food and water at all times. Animal treatment and maintenance were carried out in accordance with the guidelines established by the National Institutes of Health for the care and use of laboratory animals and were approved by the Animal Care Committee of the Kunming Medical University.

After 5 days of habituation, 18 mice were randomly assigned to one of the following groups (*n* = 6 in each group): control, ASCs, and ASCs + bFGF group. Mouse in control group was injected with 2 mL aspirated fat and 100 *μ*L medium directly. The other mouse was injected with a mixture containing 1 × 10^6^ ASCs and 2 mL of aspirated fat treated with 100 *μ*L medium (ASCs group) or 100 U bFGF (ASCs + bFGF group). The fat was injected subcutaneously into the back using a 14 G needle while the mice were manually restrained.

After 12 weeks, all mice were humanely killed, and the fat grafts were carefully dissected from their back. Each fat graft was weighed and the volume was measured by the liquid overflow method according to Ayhan et al. [11]. The survival ratio for transplanted fat was calculated by using the formula: survival volume/previous volume (2 mL) [5]. And then each fat graft was divided into two equal portions. One portion was used to determine the expression levels of VEGF, PDGF-BB, and MMP2. The other portion was placed in 4% paraformaldehyde and used for histological examination.

### 2.5. Histology and Immunohistochemistry

Paraffin-embedded fat graft histological sections (5 *μ*m) were prepared and then stained with hematoxylin and eosin using standard procedures. For immunohistochemistry, monolayer ASCs grown on glass slides or fat sections were incubated with 0.3% H_2_O_2_ for 15 min at room temperature to block endogenous peroxidase activity, followed by incubation for 2 hours in blocking solution at room temperature, and sequentially incubated with anti-CD29 (1 : 100 dilution), anti-CD44 (1 : 100 dilution), anti-CD105 (1 : 100 dilution), anti-CD34 (1 : 100 dilution), anti-CD45 (1 : 100 dilution), or anti-factor VIII (1 : 200 dilution) at 4°C overnight. After washing with PBS, the sections/slides were incubated with biotinylated goat anti-rabbit IgG for 1 hour, followed by incubation for 1 hour in an avidin-biotin-peroxidase complex solution. Visualization was done by 3,3-diaminobenzidine (DAB). Sections and slides were examined under an Olympus microscope.

Microvascular density (MVD) was measured according to Sharma's method using the following formula: Number of microvessels in ×400 grid field/Area of ocular grid (0.0625 mm^2^) [12]. Each factor VIII positive endothelial cell cluster and clearly separate from an adjacent cluster was counted as an individual blood vessel. The MVD and blood vessels in the fat grafts were estimated in five fields per section using six sections.

### 2.6. Western Blot

The fat grafts were lysised in RIPA buffer supplemented with protease inhibitor cocktail and then centrifuged at 12,000 rpm for 15 min at 4°C. Afterward, the supernatant was collected and protein concentration was estimated by BCA Protein Assay Kit. 40 *μ*g protein was loaded onto SDS-PAGE and then transferred to polyvinylidene difluoride membrane, after incubation for 2 hours at room temperature with blocking milk; membranes were then incubated with primary antibodies: anti-VEGF (1 : 1000), anti-PDGF-BB (1 : 1000), anti-MMP2 (1 : 1000), and anti-*β*-actin (1 : 2000) at 4°C overnight. After washing, the membranes were incubated with horseradish peroxidase-conjugated secondary antibody (1 : 5000) for 2 hours at room temperature. The blots were developed using an enhanced chemiluminescent assay. Band intensities were quantified using Image-Pro Plus 6.0 software (Media Cybernetics Inc., Bethesda, MD, USA).

### 2.7. Statistical Analysis

Data were presented as mean ± SD. Differences between mean values were analyzed using one-way ANOVA followed by post-hoc tests using SPSS statistical software program (SPSS Inc., Chicago, IL, USA). Statistical significance was set at *P* ≤ 0.05.

## 3. Results

### 3.1. Characterization of ASCs from Human Fat Tissues

**Figure 1:**
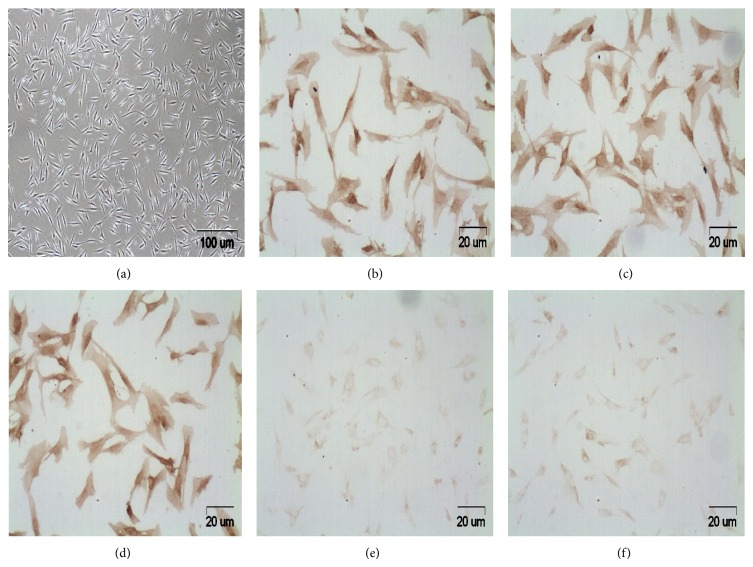
Photomicrographs of primary culture ASCs ((a), magnification ×40) and mesenchymal stem cell characteristics identified by CD29, CD44, and CD105 positive, respectively ((b)–(d), magnification ×200) and hemopoietic lineage markers CD34 and CD45 negative ((e) and (f), magnification ×200) using immunohistochemistry.

The cells isolated from human abdominal adipose tissue formed a monolayer after 7 days seeding on tissue culture plastic. Under microscopic examination (Figure 1(a)), the adherent cells displayed a fibroblast-like spindle-shaped morphology that was consistent with previous descriptions of ASCs [13]. To confirm the cells as stem cell, cells were examined by immunohistochemistry for expression of CD markers and most were found to be positive for CD29, CD44, and CD105 (Figures 1(b)–1(d)), but negative for CD34 and CD45 (Figures 1(e) and 1(f)).

### 3.2. Fat Grafts Weights and Survival Ratios

The weights and survival ratios of fat grafts are shown in Table 1. The data demonstrated that the weight and survival ratio in ASCs and ASCs + bFGF treated fat grafts were significantly elevated compared to control group. The further analysis revealed that bFGF treatment had a more pronounced beneficial effect on the fat grafts weight and survival ratio while compared with ASCs alone group.

### 3.3. Histological Evaluation

**Figure 2:**
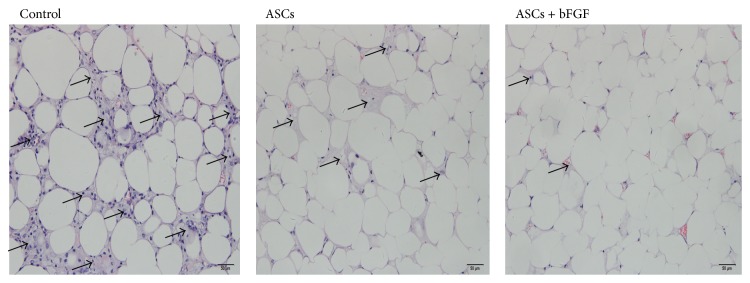
HE stain evaluation of fat grafts (magnification ×200). The control grafts exhibited excessive fibrosis (shown as ↑); in the group with ASCs, the grafts consisted predominantly of mature lipocyte and had significant lower levels of fibrosis; and bFGF ameliorated fibrosis more.

With hematoxylin and eosin (HE) staining, all grafts exhibited the histological structure of normal fat tissue; although fibrosis was observed in the three groups, the grafts mixed with ASCs had significantly lower levels of fat fibrosis and more homogeneous distribution of lipocyte (Figure 2). Moreover, the tissue harvested from ASCs + bFGF treated group exhibited modestly low fibrosis compared to ASCs alone group (Figure 2).

### 3.4. Blood Vessel Generation and MVD in Fat Grafts

**Figure 3:**
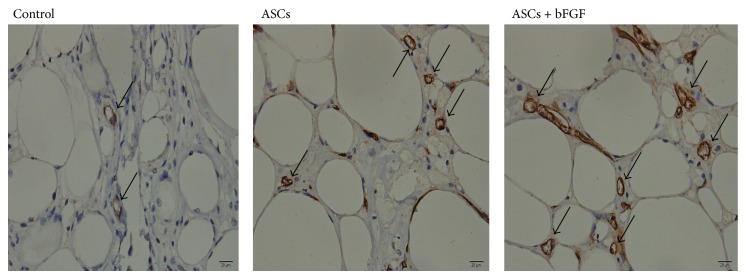
Vascularization in the fat grafts as evidenced by factor VIII immunohistochemistry (magnification ×400). Fewer factor VIII positive blood vessels were observed in control grafts while compared to ASCs group, and bFGF further promoted blood vessel generation while compared to grafts received ASCs alone.

As shown in Figure 3, newly generated blood vessels were observed in the fat grafts in the three groups, and there were well-vascularized areas evidenced by increasing expression of factor VIII in grafts received ASCs. Meanwhile, the MVD observed in the ASCs and ASCs + bFGF treated fat grafts were significantly higher than those in the control fat grafts (Table 1); also the MVD elevated moderately in ASCs + bFGF grafts while compared with ASCs alone grafts.

### 3.5. Angiogenic Factors Expression in Fat Grafts

**Figure 4:**
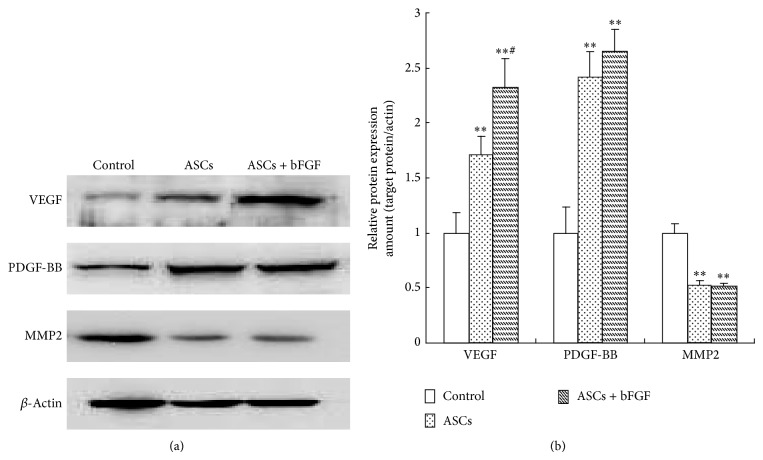
(a) Western blotting analysis of the expression of VEGF, PDGF-PP, and MMP2 in fat grafts. (b) Quantitative analysis of protein levels by densitometry. The data from western blot were normalized by taking the value of control group as 1. ^**^ *P* < 0.01 versus control group; ^#^ *P* < 0.05 versus ASCs group.

As shown in Figure 4, western blot analysis revealed a significant increase in VEGF and PDGF-BB but decrease in MMP2 protein expression in ASCs and ASCs + bFGF treated grafts compared to control, and the VEGF protein expression in ASCs + bFGF group was also higher than that observed in ASCs group; although there was a slight elevation in PDGF-BB expression in bFGF-treated fat grafts, there was no statistics difference between the bFGF + ASCs and ASCs group, and the change of MMP2 also has no statistics difference while comparing ASCs + bFGF group to ASCs group.

## 4. Discussion

Our results demonstrated bFGF treatment improves fat grafts survival and fat integration with less fibrosis. Furthermore, bFGF significantly increased the expression levels of various angiogenic factors, especially in VEGF, and promoted blood vessel generation. The findings indicated the beneficial effects of bFGF on vasculogenesis better for transplanted fat survival.

Although injective autologous fat transplantation is an ideal method for filling soft-tissue defects, the application of autologous fat transplantation is still in restrain due to the low survival rate after transplant. With the recent developments in stem cell therapy research, ASCs are transplanted into recipients along with the aspirated fat to assist graft survival. The success of ASCs relied on their proliferation and adipogenic differentiation abilities; meanwhile, various studies have shown that adequacy of angiogenesis with sufficient blood supply in transplants is required for ASCs and fat graft long-term survival [14]. However, due to the mechanical injury during the liposuction procedure, the microvessels are destroyed although the basic structure of adipose tissue can be preserved in aspirated fat [2], which makes the aspirated fat used for transplanting contain fewer microvessels; undoubtedly, the efficiency of ASCs has diminished by the relative deficiency of microvessels in transplants [1, 15]. Therefore, it remains a challenge for researchers to seek an effective solution to improve angiogenesis that finally resulted in boosting the efficiency of transplantation.

bFGF is considered a promoter of angiogenesis. Previous study has found that exogenous bFGF could enhance preadipocytes survival and vascularization in transplants [16]. Recently, Kim and colleagues demonstrated that blood vessels were significantly increased in rat infarcted hearts while they transplanted with ASCs immobilized with bFGF [17]. In this study, we examined the effects of bFGF on blood vessel generation and fat grafts survival. Similar to the traditional fat autotransplants survival ratio (~50%) [18], the present findings demonstrated that free adipose injection showed an average survival ratio of 48.2%. Not surprisingly, the use of ASCs significantly enhanced the survival of fat graft (71.1%), and the exogenous bFGF treatment had a more pronounced beneficial effect on the fat survival (81.3%). Histological examination also demonstrated bFGF improved the quality and viability of fat grafts, with slight fibrosis and more homogeneous lipocyte compared to the untreated fat and control fat. For the morphological observation of blood vessel generation, immunohistochemical staining for factor VIII (a surface antigen of vascular endothelium) has been performed. Paralleled to survival results, the transplants mixed with ASCs had a significantly higher in factor VIII positive vessel compared to control; we then measured the MVD and found that bFGF treatment achieved a better MVD than using ASCs alone, which means bFGF treatment extends the angiogenesis in fat grafts.

Angiogenesis is the sprouting of microvessels from a preexisting capillary network and the process involves a harmonized interplay between various angiogenic and antiapoptotic factors including VEGF, PDGF-BB, which may stimulate microvascular endothelial cells proliferation that promote angiogenesis, and proteases such as MMP2, which may digest extracellular matrix that hinders angiogenesis. Yi et al. showed that VEGF transfected enhances the survival of grafted fat tissue and this beneficial effect is based on induction of angiogenesis [19]. Similarly, Li et al. reported that VEGF long-time release significantly increases capillary density in fat grafts and contributes to the quality of fat grafts [20]. In the present study, we demonstrated the increased VEGF and PDGF-BB and the decreased MMP2 protein expression in fat grafts in ASCs + bFGF and ASCs alone group compared with control group, and the exogenous bFGF treatment showed a higher VEGF level compared with the same parameters in fat grafts received ASCs alone, but there were no differences in PDGF-BB and MMP2 expression between ASCs alone and ASCs + bFGF treated grafts. Indeed, bFGF increases VEGF expression but has no effect on PDGF-BB and MMP2 that was observed in our study implies that bFGF induced angiogenesis through a VEGF-dependent mechanism. Meanwhile, although the mechanism of bFGF on VEGF regulation needs more exploration, some studies that have uncovered bFGF may have modulated VEGF through ERK/AKt pathway [21]; maybe this is why the bFGF treated grafts have a higher VEGF level. Finally, it is needed to point out that some experiments have shown that MMP2 provide a favorable environment for initiation of angiogenesis especially in tumor vessel formation [22], and it seems that high MMP2 expression benefits from angiogenesis. However, Chiru and colleagues [23] reported that MPP2 play a two-sided role during the angiogenesis process, it may improve vessel generation by facilitating cell proliferation, and also, it may impede vessel generation by inducing apoptosis and breaking vessels' basal membrane. The contrary effect of MMP2 may be based on the concentration since it has been revealed that low concentration of MMP2 showed positive effect while high concentration showed an inhibitory effect [24]. Although the exact concentration of MMP2 has not been measured in this study, the western blot results demonstrated a higher level of MMP2 in control graft compared to ASCs treated graft, which suggested the negative effect of MMP2 may be a reason for the fewer vessel generation in control graft.

## 5. Conclusion

In summary, in the current study, we compared the survivals between ASCs + bFGF and ASCs alone treated fat grafts. Although there were still some limitations in this work, for example, (1) only one concentration of bFGF has been chosen, and the relations between graft survival and different bFGF concentrations have not been evaluated; and (2) VEGF silence or bFGF receptor antagonist has not been used to analyze the precise molecular mechanism of bFGF on VEGF and angiogenesis. Our results demonstrated that bFGF treatment can significantly improve the survival of transplanted fat and that beneficial effect, at least in part, attributable to its ability increase VEGF expression which eventually in favor of angiogenesis.

## Figures and Tables

**Table 1:** Evaluate the weight, survival, and MVD in different fat grafts.

Groups	Weights (g)	Survival ratios (%)	MVD
Control	0.60 ± 0.09	0.48 ± 0.04	32.00 ± 3.67
ASCs	0.82 ± 0.13^**^	0.71 ± 0.07^**^	45.6 ± 2.91^**^
ASCs + bFGF	1.06 ± 0.07^∗∗#^	0.81 ± 0.05^∗∗#^	56.01 ± 3.76^∗∗#^

^**^ *P* < 0.01 versus control group; ^#^ *P* < 0.05 versus ASCs group.
